# Measuring dissociation across adolescence and adulthood: Developing the short-form Černis Felt Sense of Anomaly scale (ČEFSA-14)

**DOI:** 10.1017/S1352465823000498

**Published:** 2023-11-06

**Authors:** Emma Černis, Bao S. Loe, Katie Lofthouse, Polly Waite, Andrew Molodynski, Anke Ehlers, Daniel Freeman

**Affiliations:** a School of Psychology, University of Birmingham, Edgbaston, Birmingham, B15 2TT; b Institute for Mental Health, University of Birmingham, Edgbaston, Birmingham, B15 2TT; c Department of Psychiatry, University of Oxford, Warneford Lane, Oxford, OX3 7JX; d The Psychometrics Centre, University of Cambridge, Cambridge Judge Business School, Trumpington Street, Cambridge CB2 1AG; e Department of Experimental Psychology, University of Oxford, New Radcliffe House, Oxford, OX2 6GG; f University of East Anglia, Norwich Medical School, Chancellors Drive, Norwich, NR4 7TJ; g Oxford Health NHS Foundation Trust, Warneford Hospital, Oxford, OX3 7JX; h Oxford Centre for Anxiety Disorders and Trauma, Department of Experimental Psychology, University of Oxford, The Old Rectory, Paradise Square, Oxford, OX1 1TW

**Keywords:** dissociation, psychometrics, measurement, adolescents, adults, felt sense of anomaly

## Abstract

**Background:**

Dissociation may be important across many mental health disorders, but has been variously conceptualised and measured. We introduced a conceptualisation of a common type of dissociative experience, ‘felt sense of anomaly’ (FSA), and developed a corresponding measure, the Černis Felt Sense of Anomaly (ČEFSA) scale.

**Aims:**

We aimed to develop a short-form version of the ČEFSA that is valid for adolescent and adult respondents.

**Method:**

Data were collected from 1031 adult NHS patients with psychosis and 932 adult and 1233 adolescent non-clinical online survey respondents. Local structural equation modelling (LSEM) was used to establish measurement invariance of items across the age range. Ant colony optimisation (ACO) was used to produce a 14-item short-form measure. Finally, the expected test score function derived from item response theory modelling guided the establishment of interpretive scoring ranges.

**Results:**

LSEM indicated 25 items of the original 35-item ČEFSA were age invariant. They were also invariant across gender and clinical status. ACO of these items produced a 14-item short-form (ČEFSA-14) with excellent psychometric properties (CFI=0.992; TLI=0.987; RMSEA=0.034; SRMR=0.017; Cronbach’s alpha=0.92). Score ranges were established based on the expected test scores at approximately 0.7, 1.25, and 2.0 theta (equivalent to standard deviations above the mean). Scores of 29 and above may indicate elevated levels of FSA-dissociation.

**Conclusions:**

The ČEFSA-14 is a psychometrically valid measure of FSA-dissociation for adolescents and adults. It can be used with clinical and non-clinical respondents. It could be used by clinicians as an initial tool to explore dissociation with their clients.

Dissociative symptoms – including highly subjective anomalous experiences of finding one’s own body unreal, memories absent, or surroundings as unexpectedly unfamiliar – are increasingly being considered from a multidimensional perspective (e.g. Černis et al., 2021; Holmes et al., 2005). In contrast to the unidimensional approach which posits that such experiences represent ‘manifestations of a single underlying process’ of dissociation (Briere et al., 2005; p.222), the multidimensional approach suggests that clusters of dissociative phenomenological experience form separable constructs. Feasibly, these constructs may have different aetiologies and require different treatment approaches (Holmes et al., 2005), and thus greater precision is required in the measurement of dissociative experiences, if we wish to disentangle the broader construct of ‘dissociation’ into its multiple dimensions.

We recently delineated one such dimension: ‘felt sense of anomaly’-type dissociation (FSA-dissociation; Černis et al., 2021). Developed *de novo* using a ‘bottom-up’, phenomenological approach, FSA-dissociation describes dissociative symptoms which have in common a subjective sense of ‘strangeness’ or anomaly, including feelings of (unexpected) unreality, unfamiliarity, automaticity, disconnection, or absence in relation to one’s body, mind, mood, perception, identity, behaviour, or external environment. In this way, FSA-dissociation overlaps phenomenologically with depersonalisation-derealisation (DPDR), since both DPDR and FSA-dissociation encompass ‘strange’ and ‘unreal’ feelings relating to the self and one’s surroundings. Recently, the two have been demonstrated to be highly correlated, but not identical (Lofthouse et al., 2023). Corresponding measures for FSA-dissociation – the Černis Felt Sense of Anomaly (ČEFSA) scale and the more general Global Felt Sense of Anomaly (GFSA) scale – were developed with a group of 8861 online survey respondents and validated with a group of 1031 patients with psychosis (Černis et al., 2021). This analysis demonstrated that the scales had highly promising psychometric properties (model fit, internal reliability, test-retest reliability, and convergent validity) in both clinical (psychosis) and general population adult groups.

However, as noted in that analysis, further development of the scale is required. In particular, the current version of the scale is not validated for use with respondents below the age of 18. This is important, given that the high-risk period for the first incidence of severe mental illness spans this divide (Kessler et al., 2007; Uhlhaas et al., 2023), and is commonly cited as occurring between mid-teens and mid-to-late twenties (e.g., 14-29 years in Ultra High Risk for psychosis (Waite et al., 2020; Yung et al., 2005); and 15-24 years for bipolar at-risk (Bechdolf et al., 2010)). Thus, research and clinical services tailored to groups spanning this nominal divide (e.g., early intervention in psychosis services; and “0 to 25 years” services) require measures that are valid for the age of the client, and using one measure – regardless of age – may be more convenient than adopting a child and adult version of the same measure. This will also permit easier comparison of a client’s scores over time if they move through the age-18 divide during the observation or treatment period.

Therefore, in the present study, we sought to improve the original 35-item ČEFSA scale in three key ways. First, we aimed to expand the utility of the scale beyond its initial validation in adults aged 18 and above by establishing its validity in an age range of 13 years and above. We also assessed its measurement invariance across key demographics. The invariance of the scale across respondent characteristics, such as gender, clinical status, and ethnicity, is important to establish, to ensure it is appropriate for as wide a population as possible. Finally, we also aimed to provide a short-form version of the scale with accompanying scoring guide to improve ease of administration and interpretation. We used three main state-of-the-art statistical approaches to realise these aims.

## Measurement invariance: multigroup confirmatory factor analyses and local structural equation modelling (LSEM)

If a scale possesses measurement invariance, it is considered to measure the same construct across specified characteristics (e.g., across ethnicity, gender, or age). This may be reflected in between-group stability of: the scale’s factor structure (configural invariance), the factor loadings of items (metric or ‘weak’ invariance), and/or item intercepts (scalar or ‘strong’ invariance). Measurement invariance is important, as it tests the assumption made when comparing groups that the latent trait has been captured as accurately, and in the same manner, for all groups (Olaru & Jankowsky, 2022). Therefore, we tested the scalar invariance of the items in the original ČEFSA to determine whether they are interpreted the same way, regardless of age, gender, or clinical status.

For categorical demographics (e.g., clinical status), we employed the conventional approach of measurement invariance based on binary grouping categories to evaluate for group differences. However, for age – a continuous variable – we used LSEM to test measurement invariance. Often, continuous variables are forced into arbitrary categories for the purposes of testing for measurement invariance (e.g., 13-18 years, 19-64 years, 65 years and above). This has several limitations. Primarily, that a great deal of information can be lost in this method, since datapoints that are close together on a continuous scale (e.g. 18 years and 19 years) may be treated as very different from each other if separated into different groups (Hildebrandt et al., 2016). LSEM instead weights observations around focal points, such that closer points are weighted most heavily, and further points least heavily – following the assumption that points (i.e. ages) that are closer together will be more similar than points (ages) that are further apart (Olaru et al., 2019). Weighting and including observations around the focal points enables an increase in the effective sample size for each focal point. Thus, by determining a narrow ‘step’ between focal points, and repeating the weighting process across the full range of the observed values, LSEM offers more precise estimates and retains far more information about the behaviour of the latent trait across the (age) range.

## Development of a short-form scale: Ant Colony Optimization (ACO)

There is no single accepted way to create a short-form version of an established measure, and all methods suffer from limitations of some kind. Olaru et al. (2019) outline three challenges. First, that selecting items based on item-level characteristics often sacrifices the quality of the scale-level properties of the measure. Second, that scale-level properties change as items are included or removed. Finally, that a suitable balance must be struck between these properties. They therefore argue that the optimal item selection method must be ‘combinatorial’ (as opposed to stepwise), and must take into account multiple scale-level properties simultaneously. Ant colony optimisation (ACO) is a method that iteratively selects combinations of items, and weights items that contribute to the quality of that selection’s scale-level properties such that they are more likely to be re-selected in the next round (Olaru et al., 2019). This method may not have the most optimal variance explained (Olaru et al., 2019), but does produce a strong model fit result and does not suffer from the ‘suppressor effect’ (highly correlated items) that methods such as regularised structural equation modelling suffers from. Therefore, ACO was used to develop a psychometrically valid short-form of the ČEFSA.

## Establishing a scoring guide: Expected test score function

An interpretive scoring guide for the new short-form version of the scale was produced by consulting the expected test score function derived from an item response theory (IRT) model. IRT allows the calculation of parameters that give further information about the performance of a scale with respect to the latent trait that the scale measures (in this case, FSA-dissociation). The latent trait is represented by values of ‘theta’ along a continuum whereby lower severity of the latent trait is indicated by lower levels of theta, and higher severity by higher theta values. Determining which total score on the scale would be expected to correspond with an ‘average’ level of theta enables the identification of score ranges that would correspond with elevated, moderately severe, or severe theta levels (i.e., FSA-dissociation levels), thus producing an illustrative scoring guide. This method has been used previously (e.g. Bird et al., 2020), and is therefore appropriate to the aims of this study.

## Method

### Participants and Procedure

To assess a broad spectrum of responses, data from three groups were combined: non-clinical adolescent, non-clinical adult, and clinical (adults with psychosis diagnoses).

Non-clinical (community) adolescent data participants were recruited via UK schools as well as social media advertising. Consent procedures also took place online (*Qualtrics*, 2020): participants aged 13 to 15 years provided assent to participate following parental informed consent, and participants aged 16 to 18 years provided informed consent. Inclusion criteria were: aged 13 to 18 years, and resident in the UK. Data were collected between 5^th^ and 25^th^ November 2021. Participants were not asked about their mental health history or status. Full detail is available in Lofthouse et al., (2023).

Non-clinical adult participants were recruited via Facebook advertisements to participate in an online cross-sectional self-report questionnaire study (Černis, Ehlers, et al., 2022). Informed consent and assessment were both carried out online using Qualtrics (*Qualtrics*, 2019). Surveys were accessible on desktop and mobile web browsers. Inclusion criteria were: age 18 or above, and usually resident in the UK. Data were collected between 30^th^ January 2019 and 25^th^ February 2019. Note that this group is distinct from that used for the original measure development by Černis et al. (2021). In the present study, only data from respondents who reported no current or previous mental health difficulties were retained.

Clinical participants were recruited by Clinical Research Network (CRN) research assistants and clinical studies officers embedded in clinical and research teams across 36 NHS trusts in England to participate in a cross-sectional self-report questionnaire study (Černis, Molodynski, et al., 2022). Informed consent was obtained by CRN staff. Inclusion criteria were: age 16 years or over, currently under the care of an NHS mental health service, with a diagnosis of non-affective psychosis, and willing and able to give informed consent to participate. Exclusion criteria were: insufficient English language to complete the questionnaires even with support, or an affective psychosis diagnosis (e.g. psychotic depression, bipolar disorder). Data were collected between 18^th^ October 2019 and 19^th^ March 2020. Note that this is the same clinical group whose data was used for scale validation by Černis et al. (2021).

Selecting participants with less than or equal to 20% missing data on the ČEFSA scale resulted in data being excluded from 211 adolescent, 30 non-clinical adult, and seven clinical participants. Thus, the final dataset included from 1233 adolescent online survey respondents, 932 adult online survey respondents, and 1031 NHS patients with non-affective psychosis diagnoses, resulting in a sample of 3196 responses for analysis. This exceeds the criteria required for structural equation modelling (Bentler & Chou, 1987; Wolf et al., 2013). Whilst guidelines regarding adequate sample size for ACO do not currently exist, this sample size was determined to be more than sufficient, given that this method has been demonstrated to cope well with major misspecifications at a sixth of the sample size (Koğar, 2022).

### Ethical Statement

Authors abided by the Ethical Principles of Psychologists and Code of Conduct as set out by the BABCP and BPS. Ethical approval was obtained for non-clinical data collection from the University of Oxford Central University Research Ethics Committee (adult study: REF: R61315/RE001; adolescent study: R71497/RE001), and for clinical data collection from the NHS London (City & East) Research Ethics Committee (REF: 19/LO/1394).

### Measures

The Černis Felt Sense of Anomaly scale (ČEFSA; Černis et al., 2021) measures dissociative experiences sharing a core phenomenological experience of a felt sense of anomaly (FSA) using 35 items. The 35 items form seven factors, each of five items. Factors are: Anomalous Experience of the Self (e.g., *“I feel like a stranger to myself”*), Anomalous Experience of the Body (e.g., *“My body feels numb”*), Anomalous Experiences of Emotion (*“I don’t fully experience emotions”*), Altered Sense of Familiarity (*“Places that I know seem unfamiliar”*), Altered Sense of Connection (*“I feel detached from what I’m doing”*), Altered Sense of Agency (*“I don’t notice how much time passes”*), and Altered Sense of Reality (*“I feel like other people aren’t real”*). Items are rated for the past two weeks on a Likert scale from 0 “never” to 4 “always”. Higher scores indicate higher levels of FSA-dissociation.

The ČEFSA has good convergent validity with the Dissociative Experiences Scale (DES-II; Carlson & Putnam, 1993) (r = 0.802, p<0.001; Černis et al., 2021), and high internal consistency (Cronbach’s alpha of 0.98 in the current participant group).

### Statistical analysis

All analyses were carried out in R version 4.2.1 (R Core Team, 2022) using the following packages: sirt (v.3.12-66; Robitzsch, 2019), lavaan (v.0.6-14; Rosseel, 2012), psych (v.2.2.9; Revelle, 2022), mirt (v.1.37.1; Chalmers, 2012). Levels of missing data were low (0.13%) and were replaced via multiple imputation using the mice package (v.3.15.0; (van Buuren & Groothuis-Oudshoorn, 2011).

#### Evaluating measurement invariance across participant characteristics

To test for measurement invariance across categorical participant characteristics (e.g., gender and clinical status), multigroup confirmatory factor analyses (CFA) were carried out and different levels of measurement invariance were compared. First, we estimated the factor structure without parameter constraints across groups (the configural model). Next, we estimated the weak (or metric) model by constraining the factor loadings to be equal across groups. Finally, we estimated the strong (scalar) model where both the factor loadings and item intercepts were constrained to be equal across groups. To determine whether there was a significant difference between the nested models, we applied the criteria outlined by Chen (2007) for large sample sizes. Following these, invariance *cannot* be assumed if there is a difference of ≥ -0.01 in CFI, ≥ 0.015 in RMSEA, or ≥ 0.030 in SRMR between models.

For the continuous variable of age, we used local structural equation modelling (LSEM). Following Olaru et al., (2019), the R package *sirt* was used to apply LSEM to the data with each age in the observed range (13 to 74 years) set as a focal point, with a bandwidth parameter (*h*) of 2. As Olaru et al., (2019) describe, the bandwidth parameter artificially inflates the sample size since the weighted samples include the observations around each focal point. This creates an ‘effective’ sample size (N_eff_) that is larger than the observed sample size. Hildebrandt et al., (2016) showed that a recommended bandwidth of 2 is sufficiently accurate to detect differences in the model while reducing the effects of noise.

In this analysis, the data were treated as continuous given that items contained five response categories (Rhemtulla et al., 2012). A maximum likelihood estimator was used, and the standard errors were estimated using a bootstrap approach. Since LSEM does not allow assessment of the effect of the continuous variable (age) on model parameters, a permutation test using 1000 permutations was carried out to test the null hypothesis that the parameters do not differ significantly across the age range (Allemand et al., 2021; Hildebrandt et al., 2016).

#### Developing a short-form version of the ČEFSA

After the analysis of measurement invariance, the ant colony optimisation (ACO) was used to select the resulting items for a short-form version of the ČEFSA scale. The method used in this study follows the tutorial outlined by Olaru et al. (2019), including the advice to compare results ‘across several runs of the algorithm’ ‘in order to approach optimal solutions’ (p.403). The method outlined in this tutorial requires manual specification of the number of items to be retained by the ACO analysis. A priori discussion between EČ, BSL, and DF reached the conclusion that, if feasible following rejection of non-invariant items, then a 14-item version of the scale (two items in each of the seven factors) would be a good compromise between scale length (aiming for fewer than twenty items) and phenomenological breadth (encompassing the fullest possible range of FSA-dissociative experiences).

To ensure that the findings are robust, ACO was performed 10 times with 30 iterations and 50 ants per run, and the best fitting result across multiple runs were assessed against the optimal model fit criteria of CFI > 0.95 and RMSEA < 0.06 (Hu & Bentler, 1999).

We randomly split the dataset equally into two subsamples (training and validation) to avoid overfitting and to ensure the robustness of the item selection procedure. In the first step, we employed the ACO algorithm on the training sample to identify the best solution. In the second step, we conducted further analysis on the recommended solution using the validation sample. Specifically, we estimated the correlated factor structure of the best solution using CFA on the validation sample to evaluate the robustness of the model fit. We then performed multi-group CFA across the two subsamples to ensure parameter equivalence of the model.

#### Developing a scoring guide for the short-form version of the ČEFSA

Finally, the expected test score function was calculated, derived from the higher order factor of a second-order IRT model. This type of IRT model is mathematically equivalent to the Testlet IRT model and is a restricted version of the bi-factor model, which allows for the examination of the higher order factor based on the shared variance among multiple primary constructs (Rijmen, 2009).

Using IRT modelling to obtain the expected test function enables interpretative score guides to be established at different points across the severity spectrum (e.g. Bird et al., 2020). To establish our scoring guide, the theta range between the minimum and maximum possible test score was considered, and expected test scores at various theta values above average were inspected. The aim was to create four approximately even scoring ranges with boundaries reflective of relative severity on the basis of the spread of expected test scores (i.e., standard deviations above average)..

## Results

### Participant characteristics

The mean age of the whole group was 34.9 years (range=13-74; SD=18.6). The group comprised mainly of female (57.73%) and White (82.57%) participants (Table 1). However, there was significant heterogeneity between participant group types, with significant differences between all groups for age, gender distribution, and ČEFSA mean scores, and a significant difference between clinical and non-clinical participants with respect to ethnicity distribution.

### Measurement invariance (MI)

Due to relatively low numbers of participants in ethnic groups other than White, it was not possible to carry out a test of measurement invariance across ethnicity. Therefore, measurement invariance was tested across gender, clinical status, and age. Within the category of gender, too few participants identified as ‘other’ (n = 163 [5.10%]) and therefore MI was tested only between female and male respondents.

#### Categorical participant characteristics (gender, participant group)

Indicating that CFA was appropriate in this dataset, the Kaiser-Meyer-Olkin value was 0.98, and Bartlett’s test of sphericity was significant (Χ^2^=5608.85, p<0.001, df=595).

Model fit statistics for a correlated seven-factor structure of the scale data for all participants were adequate given the large sample size (Chi square=5041.58, df=553, p<0.001, CFI=0.929, TLI=0.923, RMSEA=0.051, SRMR=0.043).

#### Invariance across participant groups

The configural model (see Table 2 for all model fit statistics) showed good model fit, indicating the factor structure is invariant across patients with psychosis diagnoses, and adult and adolescent online respondents.

Next, weak (metric) and strong (scalar) models were estimated (Table 2). However, ten items were found to have sufficiently large item means that they were affecting model fit comparison results. Since there is no accepted convention for dealing with invariant items (Putnick & Bornstein, 2016), we opted to discard these items in order to achieve MI at the scalar level. Therefore, these models are based on a 25-item version of the ČEFSA (Supplementary Material). (Note that, when tested, the model fit for these 25-items was found to be superior to the full 35-item scale: Chi square=1815.992, df=300, p<0.001, CFI=0.964, TLI=0.957, RMSEA=0.0439, SRMR=0.0272).

Comparison of model fit statistics against the aforementioned criteria (see Statistical Analysis) indicated that the differences between the weak and strong models were sufficiently small to indicate item invariance across participant groups. Further, the strong model had the lowest BIC value of the three models, indicating that scalar invariance can be assumed.

#### Invariance across gender

The same reduced subset of 25 items used for the analysis of participant groups was used to assess configural, weak, and strong levels of MI across male and female gender (Table 2). Again, the magnitude of the differences between weak and strong model indices indicate invariance across gender groups, as does the small BIC value for the strong model relative to the other nested models.

#### Invariance across age

The average weighted sample size across the LSEMs estimated at each age in the configural model was N_eff_ = 811.108 (min=216.258 (age 74); max=1401.994 (age 17).

Measurement invariance of the 25 items across the age range was assessed at configural (CFI=0.956, RMSEA=0.054, SRMR=0.032), weak (CFI=0.956, RMSEA=0.052, SRMR=0.037), and strong (CFI=0.955, RMSEA=0.052, SRMR=0.037) levels (Table 2). Again, the magnitude of differences in the fit indices between models indicates measurement invariance at the strong level: i.e., item intercepts can be considered invariant across age. (For graphs of individual item intercepts over the age range, see Supplementary Material).

**Figure 1:**
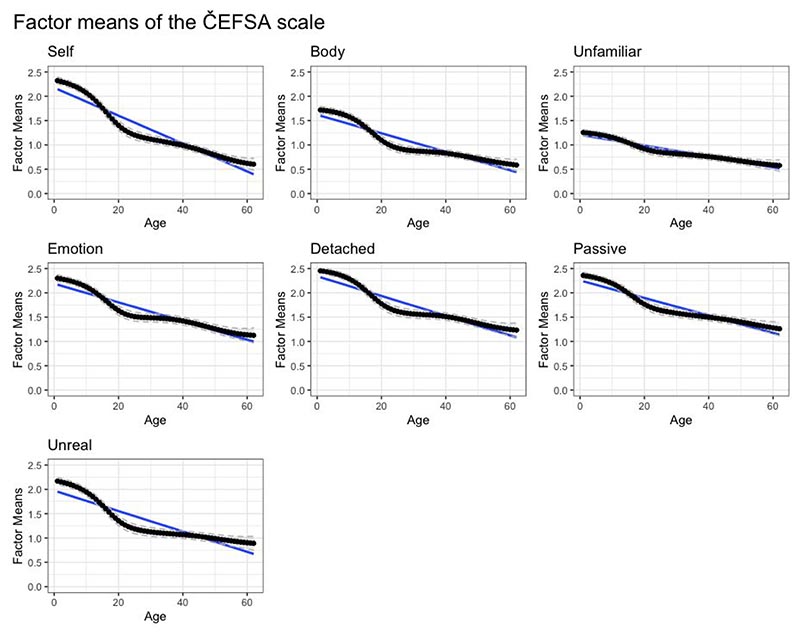
Factor means of the 25-item version of the ČEFSA scale Solid line shows point estimates at each age point. The dashed lines represent the 95% confidence interval for each point estimate. Blue lines show the linear approximation. Key: "Self" - Anomalous Experience of the Self factor "Body" - Anomalous Experience of the Body factor "Unfamiliar" - Altered Sense of Familiarity factor "Emotion" - Anomalous Experience of Emotion factor "Detached" - Altered Sense of Connection factor "Passive" - Altered Sense of Agency factor "Unreal" - Altered Sense of Reality factor

Since the scale is invariant at the item level, factor means were calculated to identify any trends across the age range. This analysis revealed that factor means did not remain stable over age (Figure 1). Rather, there was a steep decline in mean scores as age increased. Between mid-to-late-teens and approximately 30 years of age, factor means dropped by approximately one standard deviation over the 15 years. This trend continued at a shallower rate from 30 years of age to the end of the sampled range: factor means dropped by approximately half a standard deviation over this 30-year span. This pattern was true for all seven factors, but particularly pronounced in the ‘Altered Sense of Reality’ (‘Unreal’) factor (Figure 1). (Visual inspection of the mean raw scores for each factor across age (see Supplementary Material) confirmed this pattern was not an artefact of LSEM sampling).

Note, however, that caution is required when interpreting results around 65 years and above (and at the lowest end of the observed age-range), since the lack of sample size availability reduces the fitness of the model at these extremes. Illustrating this, plotting the model fit indices showed a stable trend as age increased, until approximately age 65 years, when drop-off of fitness rapidly declined (see Supplementary Material).

Confirming the pattern of each factor showing a decline in factor mean score as age increased, the results of the permutation test demonstrated a significant difference between observed and permuted trends, i.e., that the null hypothesis must be rejected (Table 3). The permutation test included 62 focal points for age.

### Development of the short-form ČEFSA (ČEFSA-14)

The final item set selected by ACO is shown in the Appendix. Two items were selected from each of the seven factors to produce a multifactorial 14-item scale.

The final 14 item scale achieved the optimal fit criteria. The results showed excellent model fit in both the training (Chi square=6050.17; df=56; p=0.003; CFI=0.995; TLI=0.992; RMSEA=0.026; SRMR=0.013) and validation (Chi square=6334.76; df=91; p<0.001; CFI=0.992; TLI=0.987; RMSEA=0.034; SRMR=0.017) sample. Cronbach’s alpha was excellent (0.92) in this sample. Inspection of model fit statistics for the configural, metric, and scalar models of the short-form scale indicated that it is invariant at the scalar level (CFIΔ<0.001; RMSEAΔ<0.001 [scalar versus metric]; CFIΔ<0.001; RMSEAΔ=0.001 [metric versus configural]). For factor loadings, see Supplementary Material.

### Establishing a scoring guide for the ČEFSA-14

**Figure 2:**
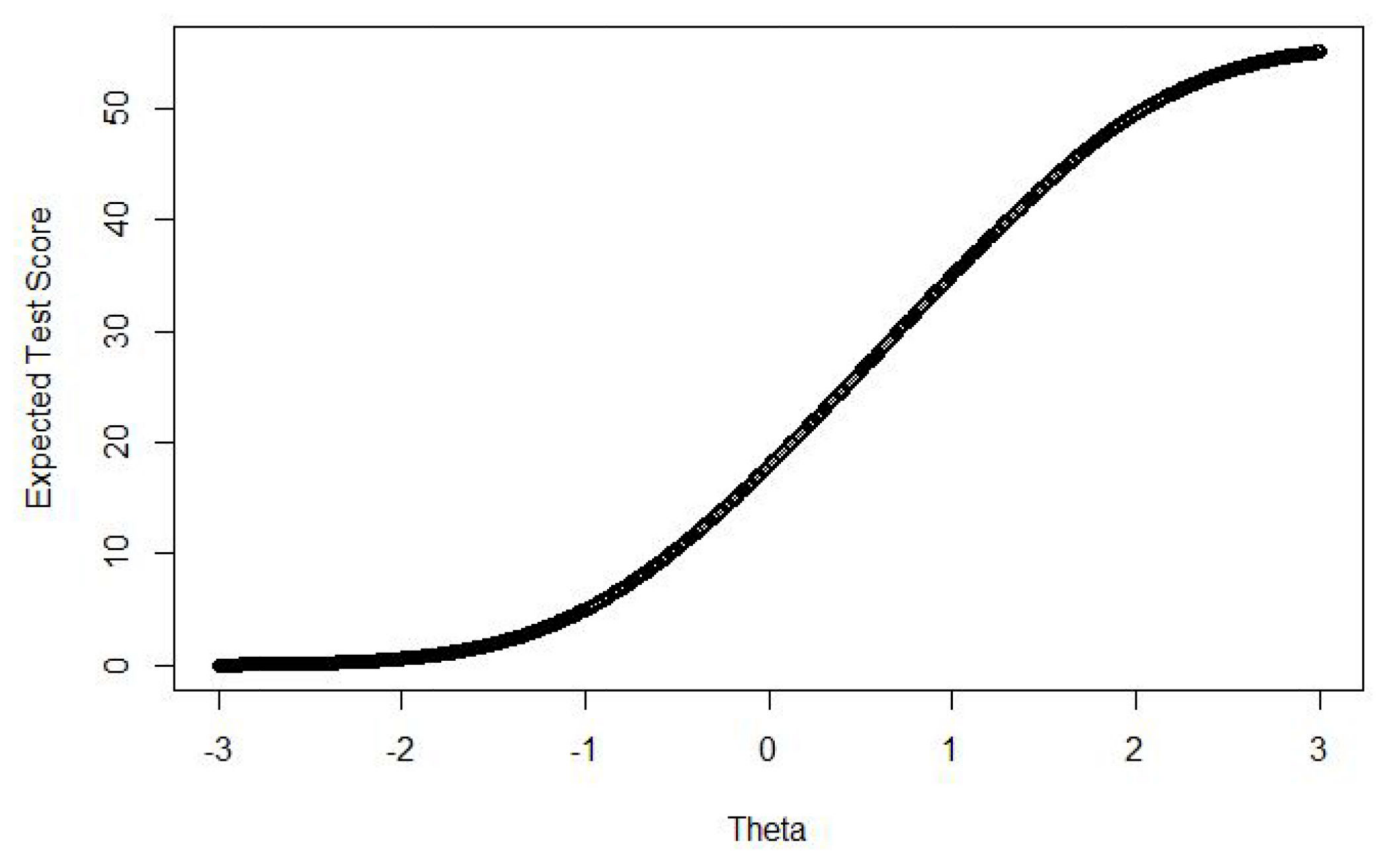
Plot of expected test score (on the 14-item ČEFSA) across the range of theta (i.e., FSA-dissociation severity).

The second-order factor IRT model showed good fit to the data (CFI=0.992; TLI=0.987; RMESA=0.0354; SRMR=0.172), indicating that calculating a total score from the 14-item ČEFSA scale is appropriate and that measurement invariance assessment for categorical variables could proceed. Using the coefficients from this model, expected test score was estimated for the full range of theta (FSA-dissociation severity) (Figure 2). This indicated that an average level of FSA-dissociation (i.e., theta=0) would correspond to a score of 17.96 out of a maximum of 56 on the short-form ČEFSA. The expected score would be 26.42 at 0.5 SD above the average (theta=0.5), 35.07 at 1 SD (theta=1), 43.07 at 1.5 SD, 49.55 at 2 SD, 53.36 at 2.5 SD, and 55.11 at 3 SD. This latter score indicates that the maximum score on the short-form ČEFSA reflects levels of FSA-dissociation approximately three standard deviations above average levels.

The correlation of the raw scores with the general factor scores from the IRT model were very high (0.978), indicating that it was appropriate to use expected test scores to establish scoring guidance. Therefore, we developed interpretive score guides (Table 4). Following Bird et al., (2020), we set the ‘high’ or ‘severe’ range to incorporate test scores where dissociation is two standard deviations above the mean (i.e. theta=2.0), and the first clinically significant range (i.e. the end of the ‘average’ range) to begin at theta=0.7. The expected test score at theta=2.0 is 49.55, thus, a scoring boundary of 49 and above was set for ‘severe’. The expected test score at theta=0.7 was 29.91, thus a scoring boundary of 28 and below was set for ‘average’.

The range of scores between 0.7 and 2.0 theta were then divided equally to produce an ‘elevated’ and ‘moderately severe’ range. This resulted in a boundary at approximately theta=1.25, or an expected test score of 39.15: therefore, a score of 38 was judged to be the highest possible score of the ‘elevated’ category, and 39 the lowest boundary of the ‘moderately severe’ category. Table 4 also reports the proportions of these four categories in each of the participant groups.

## Discussion

The precise measurement of dissociative phenomena is important. Conceptual confusion has been a key barrier to progress in this area. Here, we present the ČEFSA-14: a brief and psychometrically robust measure of a specific subtype of dissociative experience (felt sense of anomaly-type dissociation [FSA-dissociation]).

A key advantage of this short-form measure is its measure invariance. This means that there are no systematic differences in the way the ČEFSA-14 measures FSA-dissociation across gender (male/female), or clinical status (psychosis diagnosis/general population). Crucially, the scale is also invariant across age over the range of 13 years to approximately 65 years.

Although participants slightly older than this were included in our analyses, caution is required at this upper age limit due to the rapid decline in model fit above 65 years resulting from insufficient data from older adults in our group. Nevertheless, this property of the ČEFSA-14 makes it a valuable tool for investigating dissociative experiences across the aforementioned traditional (age 18 years) boundary between ‘child and adolescent’ and ‘adult’ groups. This confers significant benefits for research studies and healthcare services serving groups that span this divide, and in contexts where a longer-term course of work may mean that the respondent crosses this age-boundary during the treatment or measurement period.

To clarify the benefits and limitations of both versions (the original 35- and short-form 14-item) of the ČEFSA scale, our analysis suggests that whilst the original 35-item version of the ČEFSA has been demonstrated to be psychometrically valid for ages 18 and above (Černis et al., 2021), it is not as psychometrically robust as the interim 25-item model (Supplementary Material) presented here, since ten items in the original ČEFSA may be interpreted differently by different genders, ages, and by people with different clinical statuses. The original version is therefore a good option where the aim is to explore in further detail, with an adult respondent, the nature of their dissociative experiences, without the need to interpret their value score on the measure. Adding to the utility of the original scale as a tool to explore clients’ phenomenological experiences, the factors may be treated as separable constructs within the original version of the scale, whilst in the short-form, they have not been validated in this way. The short-form version presented here may therefore be most appropriate to use with younger ages (i.e., from 13 upwards), in services or studies with age ranges spanning 18 years, or where it may be helpful to interpret the sum score (e.g., in clinical research or audit).

However, it is also important to note an interesting finding raised by our age invariance analysis: that age is a moderating factor of dissociation across time. It was a common pattern for FSA-dissociation to be higher at younger ages, dropping rapidly between mid-teens and early-thirties, before declining more slowly from this point until the upper age limit of the scale. This finding underscores the importance of validating the ČEFSA measure below age 18 years, and reflects previous similar results. For example, Ross et al., (1989) report that in a group of 168 school and college students, Dissociative Experiences Scale (DES; Carlson & Putnam, 1993) scores were higher in adolescents and appeared to decline with increasing age. More recently, Tolmunen et al., (2007) reported a comparable finding using the adolescent version of the DES, noting that ‘although statistically significant, the difference may not have clinical relevance […] our findings may reflect [normative] development’ (p.616).

For this reason, and due to the method used to develop them, the interpretive scoring guides for the ČEFSA-14 presented here should be used with clinical judgement, and should not be referred to as a ‘clinical cut-off’. Indeed, they may be better understood as a preliminary guide to be updated in future^1^ following the collection of data where dissociation levels have been clinically-validated (e.g. via use of the Structured Clinical Interview for DSM-IV Dissociative Disorders (SCID-D); Steinberg, 1994). Using high-quality data of this kind to establish a more robust scoring guide is the next step in validating this measure. Given the age-related trend, future further validation of this scale may also seek to include longitudinal data to determine how scoring ranges may be interpreted intra-individually over time.

There are further limitations to the work presented here. We did not have sufficient data to test for invariance across ethnicity, nor to include the ‘other’ gender group in our test for invariance across gender. Therefore, data with adequate representation of ethnic and gender minorities are also required for the next step in further developing and validating the ČEFSA-14 measure. As noted above, there were also less data available at the upper end of the age range, meaning that further data collection is required to confidently validate the observed continued downward trend in frequency of dissociative experiences in older adults. Additionally, future research may wish to explore the expression of dissociation at this age, to understand whether there are any qualitative differences, as well as quantitative, in comparison with dissociation in adults and adolescents.

Nonetheless, the brief scale presented here offers a valid and effective way for researchers to measure FSA-dissociation across the age-18 divide, and for clinicians to interpret the levels of such phenomena in their clients whilst opening up conversation about these difficult-to-describe, distressing experiences.

## Supplementary Material

Supplementary Materials

## Figures and Tables

**Table 1:** Demographic details and difference tests for the final group (n=3196)

			Subgroup			Tests for significant difference between subgroups
		Whole group (n=3196)	*Non-clinical adolescents* (n=1233)	*Non-clinical adults* (n=932)	*Clinical adults* (n=1031)	
		n (%)				
**Gender**	*Male*	1133 (35.45)	278 (22.55)	136 (14.59)	719 (69.74)	*F=451.1, p<0.05
	*Female*	1845 (57.73)	750 (60.83)	792 (84.98)	303 (29.39)	
	*Other*	163 (5.10)	157 (12.73)	1 (0.11)	5 (0.48)	
**Ethnicity**	*Asian (any background)*	166 (5.19)	63 (5.11)	5 (0.54)	98 (9.51)	**F=96.62, p<0.05
	*Black (any background)*	198 (6.20)	18 (1.46)	4 (0.43)	176 (17.07)	
	*Mixed or multiple heritage*	139 (4.35)	82 (6.65)	13 (1.39)	44 (4.27)	
	*White (any background)*	2639 (82.57)	1055 (85.56)	895 (96.03)	689 (66.83)	
	*Other*	28 (0.88)	6 (0.49)	4 (0.43)	18 (1.75)	
		**Mean (SD)**				
**Age (years)**		34.85 (18.58)	15.87 (1.29)	52.56 (13.18)	41.54 (12.32)	*F=3898, p<0.05
**ČEFSA score**		47.36 (33.45)	68.14 (31.27)	27.96 (22.17)	39.54 (30.48)	*F=573, p<0.05

*Key*: *ČEFSA - The Černis Felt Sense of Anomaly scale*

*Tukey HSD test results*:

* *All three groups differ significantly from each other*.

** *The clinical group differs significantly from the two non-clinical groups, but the non-clinical groups do not differ significantly from each other*.

**Table 2:** Showing the model fit indices for all invariance models for the demographics variables.

Categorical Demographic Variable	Model	Model comparison	K	Chi square	df	AIC	BIC	CFI	RMSEA	SRMR
**Participant group** *(adult non-clinical; adolescent non-clinical; NHS patient with psychosis)*	Configural	-	288	2543.06	762	191928.80	193669.01	0.954	0.047	0.034
	Weak (metric)*	Weak vs Configural	288	2692.87	798	192020.49	193543.17	0.951 (Δ=-0.003)	0.048 (Δ=0.001)	0.043 (Δ=0.009)
	Strong (scalar)*	Strong vs Weak	302	2925.71	834	192218.43	193523.58	0.945 (Δ=-0.006)	0.049 (Δ=0.001)	0.044 (Δ=0.001)
**Gender** *(male, female)*	Configural*	-	192	1954.55	508	182781.88	183928.13	0.963	0.044	0.029
	Weak (metric)*	Weak vs Configural	192	1988.20	526	182790.50	183799.28	0.963 (Δ<0.001)	0.044 (Δ<0.001)	0.030 (Δ=0.001)
	Strong (scalar)*	Strong vs Weak	199	2041.13	544	182771.88	183703.21	0.961 (Δ=-0.002)	0.044 (Δ<0.000)	0.030 (Δ<0.000)
**Age**	Configural	-	-	-	-	-	-	0.956	0.054	0.032
	Weak (metric)	Weak vs Configural	-	-	-	-	-	0.956 (Δ<0.001)	0.052 (Δ=-0.002)	0.037 (Δ=-0.005)
	Strong (scalar)	Strong vs Weak	-	-	-	-	-	0.955 (Δ=-0.001)	0.052 (Δ<0.001)	0.037 (Δ<0.001)

* *Note: These models are based on retaining only 25 of the original 35 ČEFSA items*.

*Key: K: number of parameters; df: degrees of freedom; AIC: Akaike Information Criteria; BIC: Bayesian Information Criteria: CFI: Comparative Fit Index; RMSEA: Root Mean Square Error of Approximation; SRMR: Standardised Root Mean Square Residual*.

**Table 3:** Permutation test for LSEM (conducted with h=2).

Factor	Loadings		
	M	SD	p(SD)
Anomalous Experience of the Self	1.514	0.609	< 0.001
Anomalous Experience of the Body	1.183	0.413	< 0.001
Altered Sense of Familiarity	0.952	0.229	< 0.001
Anomalous Experience of Emotion	1.751	0.410	< 0.001
Altered Sense of Connection	1.878	0.438	< 0.001
Altered Sense of Agency	1.841	0.387	< 0.001
Altered Sense of Reality	1.504	0.488	< 0.001

*Key*: *M = weighted average of the parameter function; SD = test statistic of the permutation test; p(SD) = p value of the permutation test*.

**Table 4:** Interpretive score ranges for the 14-item short-form version of the Černis Felt Sense of Anomaly scale (ČEFSA-14) sum score.

Category	Score range	Approximate theta range	Whole group n (%)	Non-clinical adolescents n (%)	Non-clinical adults n (%)	Clinical adults n (%)
Average	0 - 28	< 0.7	2253 (70.5)	583 (47.3)	874 (93.8)	796 (77.2)
Elevated	29 - 38	0.7 < 1.25	542 (17.0)	368 (29.8)	52 (5.6)	122 (11.8)
Moderately Severe	39 - 48	1.25 < 2.0	276 (8.6)	226 (18.3)	6 (0.6)	44 (4.3)
Severe	49 - 56	> 2.0	57 (1.8)	49 (4.0)	0 (0)	8 (0.8)

## Data Availability

The authors do not have participant consent to share the data collected in this study.

## Footnotes

1 To easily communicate updates to the ČEFSA scale and scoring guide, we have created an Open Science Framework page. Please see https://osf.io/t2re7/ for the most up-to-date information.
