# Supplementary material for: Measuring dissociation across adolescence and adulthood: Developing the short-form Černis Felt Sense of Anomaly scale (ČEFSA-14)
Source: Behav Cogn Psychother. Author manuscript; Available in PMC 2024 Mar 1. (PMC7615643; doi:10.1017/S1352465823000498)
Supplement: Supplementary Materials [file EMS189333-supplement-Supplementary_Materials.pdf]

APPENDIX

Černis Felt Sense of Anomaly scale: Short-form (ČEFSA-14).

Please read the following items and rate how often you have experienced these over the past **TWO WEEKS** using the following rating:

0	1	2	3	4
Never	Rarely	Sometimes	Often	Always

Please note that this should NOT be whilst under the influence of drugs, alcohol or legal highs.

		Never	Rarely	Sometimes	Often	Always
1.	I don't fully experience emotions.	0	1	2	3	4
2.	I feel disconnected from the world around me.	0	1	2	3	4
3.	I'm absorbed in my own world and don't notice what is happening around me.	0	1	2	3	4
4.	My personality changes seemingly at random.	0	1	2	3	4
5.	I feel disconnected from other people.	0	1	2	3	4
6.	I find myself drifting off into my own world when I'm with others.	0	1	2	3	4
7.	My body (or parts of it) feels unreal or strange.	0	1	2	3	4
8.	I feel detached from my emotions.	0	1	2	3	4
9.	I act like someone else without meaning to.	0	1	2	3	4
10.	People I know seem unfamiliar.	0	1	2	3	4
11.	I feel as though other people stop existing when I can't see them.	0	1	2	3	4
12.	My body feels numb.	0	1	2	3	4
13.	Things I've done many times before seem new or unfamiliar.	0	1	2	3	4
14.	I feel like an alien or a ghost.	0	1	2	3	4

Score range (total score)	Category
0 - 28	Average
29 - 38	Elevated
39 - 48	Moderately Severe
49 - 56	Severe

0	1	2	3	4
Never	Rarely	Sometimes	Often	Always

		Never	Rarely	Sometimes	Often	Always
1.	I don't fully experience emotions.	0	1	2	3	4
2.	I feel disconnected from the world around me.	0	1	2	3	4
3.	I'm absorbed in my own world and don't notice what is happening around me.	0	1	2	3	4
4.	My personality changes seemingly at random.	0	1	2	3	4
5.	I feel disconnected from other people.	0	1	2	3	4
6.	I find myself drifting off into my own world when I'm with others.	0	1	2	3	4
7.	My body (or parts of it) feels unreal or strange.	0	1	2	3	4
8.	I feel detached from my emotions.	0	1	2	3	4
9.	I act like someone else without meaning to.	0	1	2	3	4
10.	People I know seem unfamiliar.	0	1	2	3	4
11.	I feel as though other people stop existing when I can't see them.	0	1	2	3	4
12.	My body feels numb.	0	1	2	3	4
13.	Things I've done many times before seem new or unfamiliar.	0	1	2	3	4
14.	I feel like an alien or a ghost.	0	1	2	3	4

Score range (total score)	Category
0 - 28	Average
29 - 38	Elevated
39 - 48	Moderately Severe
49 - 56	Severe

Measuring dissociation across adolescence and adulthood: Developing the short-form Černis Felt Sense of Anomaly scale (ČEFSA-14):

SUPPLEMENTARY MATERIALS

A. 25-item 'update' version of the ČEFSA, with only measurement invariant items (age, gender, clinical status) retained from the original 35-item version:

Please read the following items and rate how often you have experienced these over the past **TWO WEEKS** using the following rating:

0	1	2	3	4
Never	Rarely	Sometimes	Often	Always

Please note that this should NOT be whilst under the influence of drugs, alcohol or legal highs.

I feel detached from my physical body (or parts of it).
Places that I know seem unfamiliar.
I don't fully experience emotions.
I feel disconnected from the world around me.
I'm absorbed in my own world and don't notice what is happening around me.
My personality changes seemingly at random.
Familiar sights, smells (etc.) feel unfamiliar to me.
I can't feel emotions
I feel disconnected from other people.
I find myself drifting off into my own world when I'm with others.
The world seems like it is fake.
I feel like I don't have a personality
My body (or parts of it) feels unreal or strange.
I feel detached from my emotions.
I don't notice how much time passes.
The world around me seems unreal.
I act like someone else without meaning to.
My body feels like it's not under my control.
People I know seem unfamiliar.
I feel disconnected from my emotions.
I feel as though other people stop existing when I can't see them.
My body feels numb.
Things I've done many times before seem new or unfamiliar.
I feel detached from what I'm doing.
I feel like an alien or a ghost.

Items discarded from the original version:

- I feel like a stranger to myself.
- I feel like other people aren't real.
- My body (or parts of it) feels like it doesn't belong to me.
- People around me seem different or altered.
- I feel as if I'm experiencing life from very far away.
- The things happening around me seem unreal to me – like a dream or a movie.
- I lose track of my surroundings.
- I feel like I'm more than one person.
- My emotions don't seem real.
- I freeze, unable to do anything.

B. Item intercepts across age (25 items)

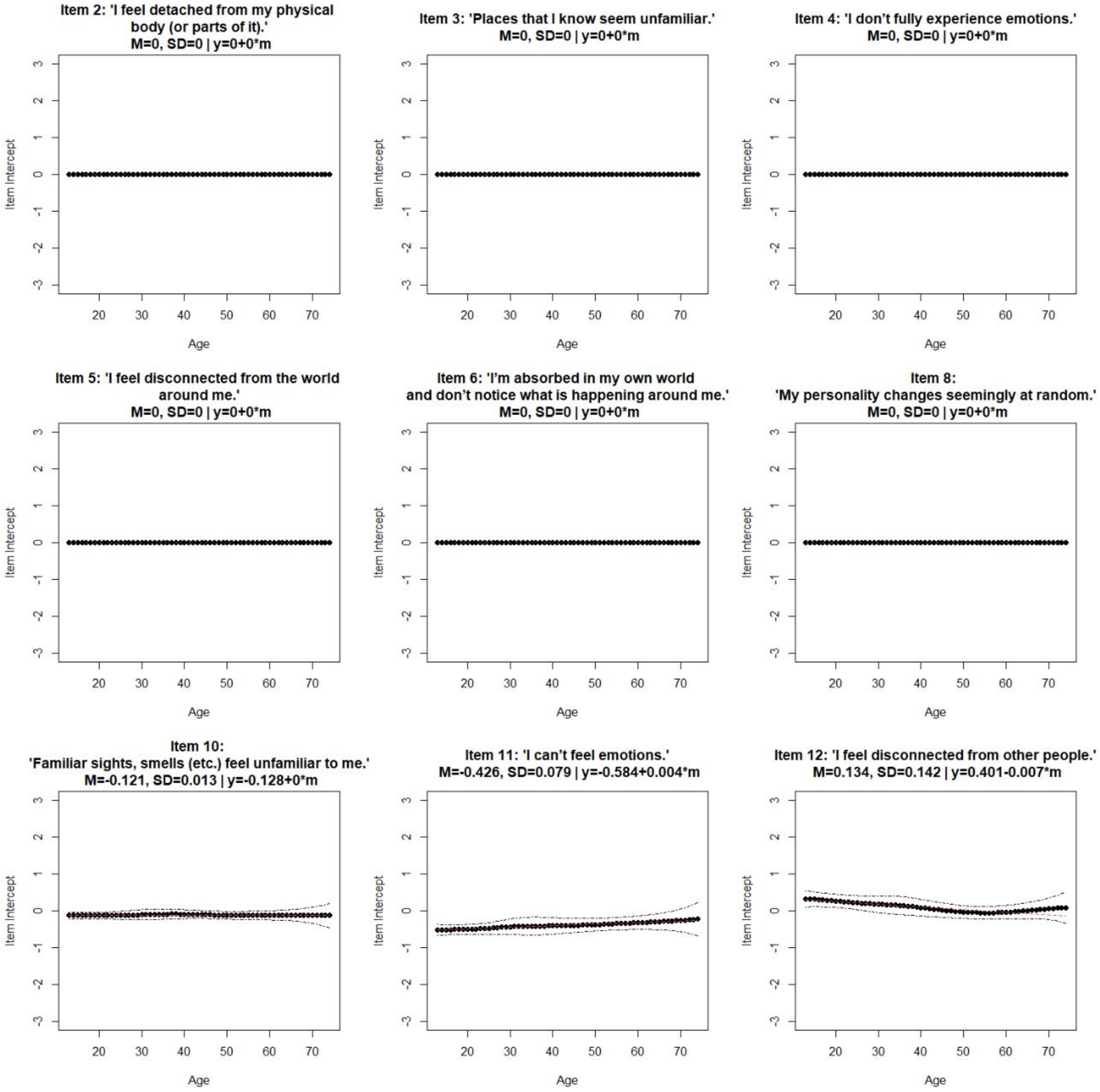

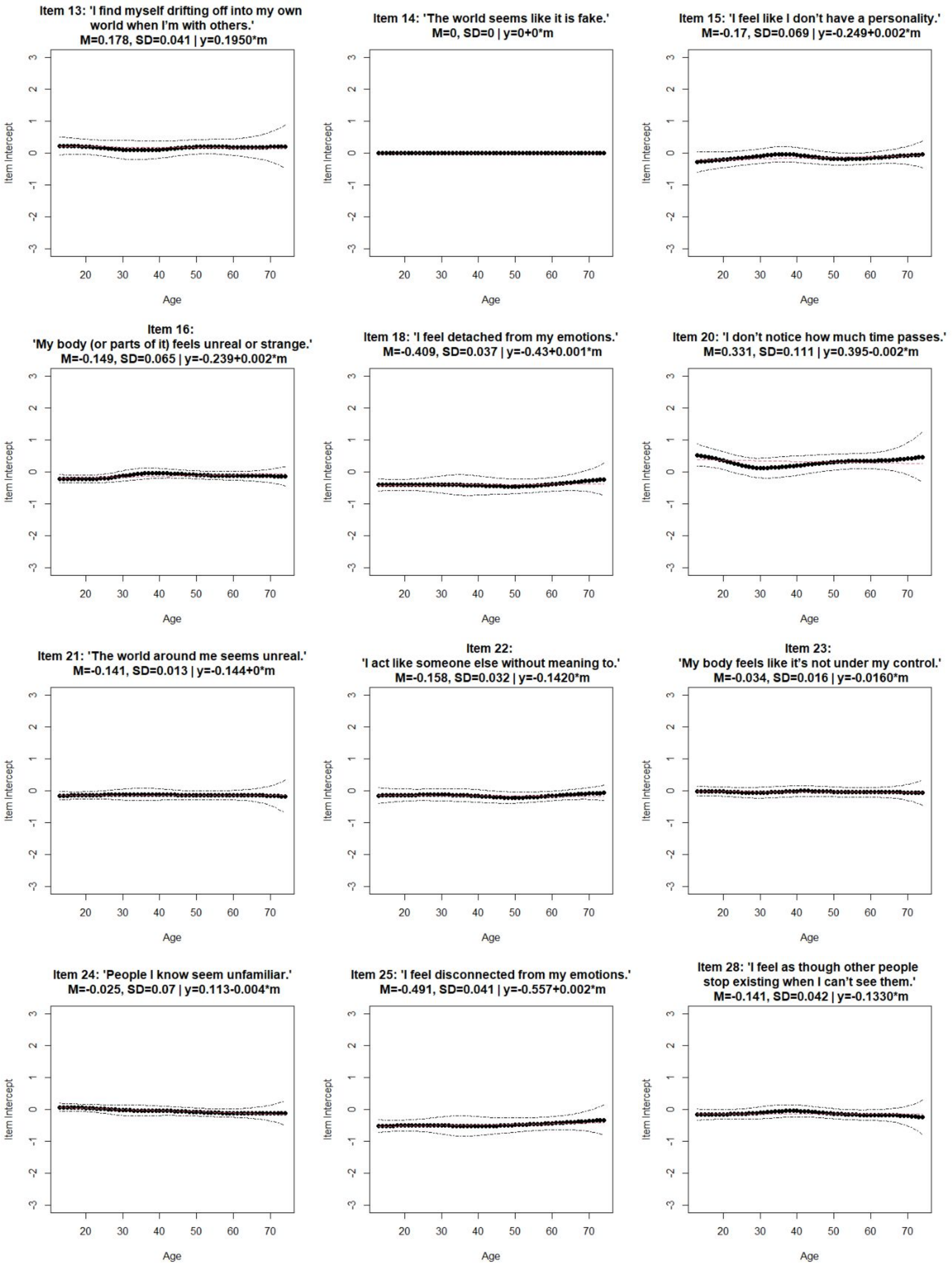
Item 13: 'I find myself drifting off into my own world when I'm with others.'
M=0.178, SD=0.041 | y=0.1950*m
Item 14: 'The world seems like it is fake.'
M=0, SD=0 | y=0+0*m
Item 15: 'I feel like I don't have a personality.'
M=-0.17, SD=0.069 | y=-0.249+0.002*m
Item 16: 'My body (or parts of it) feels unreal or strange.'
M=-0.149, SD=0.065 | y=-0.239+0.002*m
Item 18: 'I feel detached from my emotions.'
M=-0.409, SD=0.037 | y=-0.43+0.001*m
Item 20: 'I don't notice how much time passes.'
M=0.331, SD=0.111 | y=0.395-0.002*m
Item 21: 'The world around me seems unreal.'
M=-0.141, SD=0.013 | y=-0.144+0*m
Item 22: 'I act like someone else without meaning to.'
M=-0.158, SD=0.032 | y=-0.1420*m
Item 23: 'My body feels like it's not under my control.'
M=-0.034, SD=0.016 | y=-0.0160*m
Item 24: 'People I know seem unfamiliar.'
M=-0.025, SD=0.07 | y=0.113-0.004*m
Item 25: 'I feel disconnected from my emotions.'
M=-0.491, SD=0.041 | y=-0.557+0.002*m
Item 28: 'I feel as though other people stop existing when I can't see them.'
M=-0.141, SD=0.042 | y=-0.1330*m
Item Intercept
Age

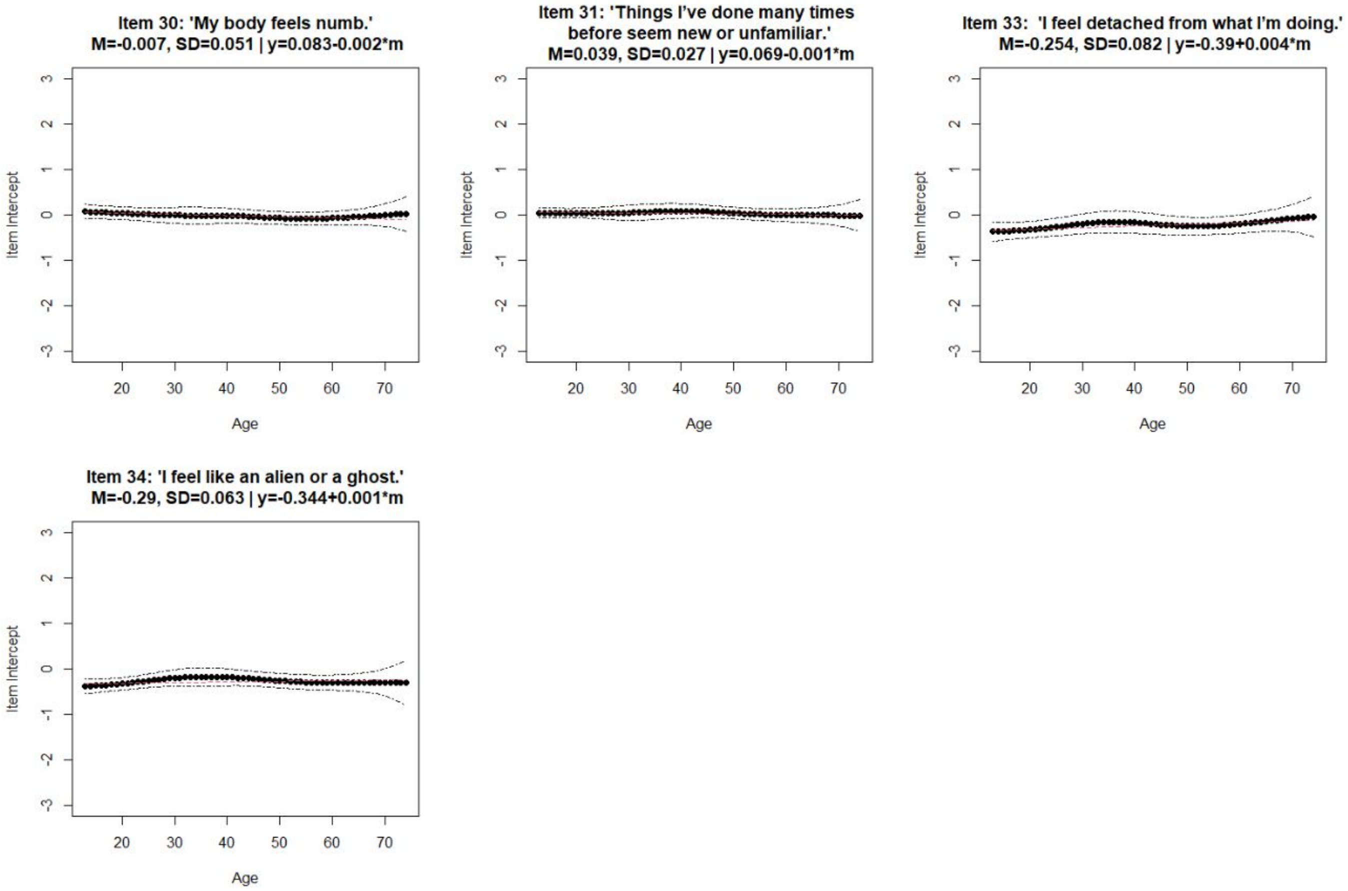

C. Mean raw scores for each factor across age (25 items)

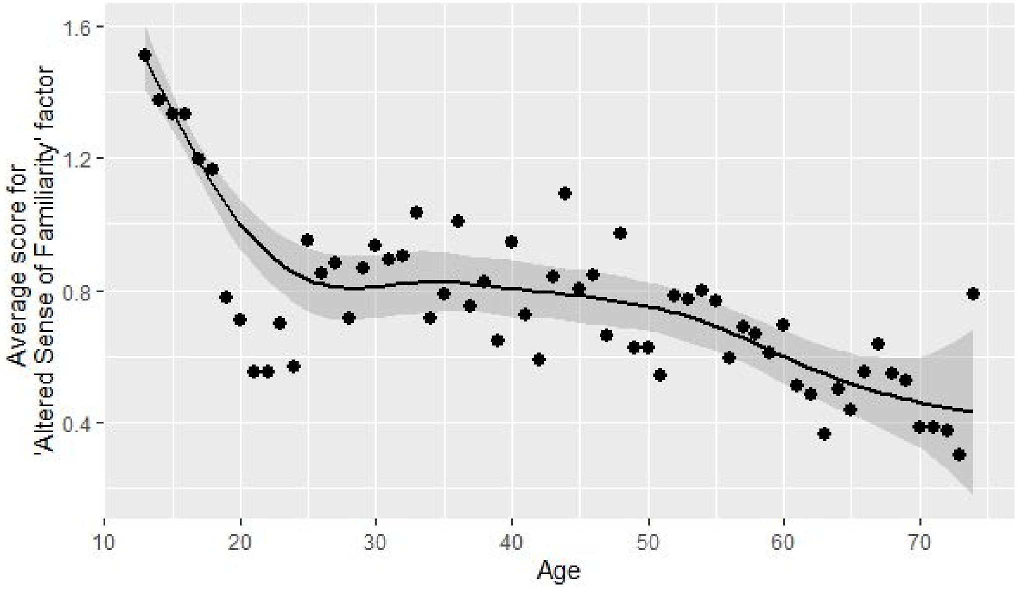

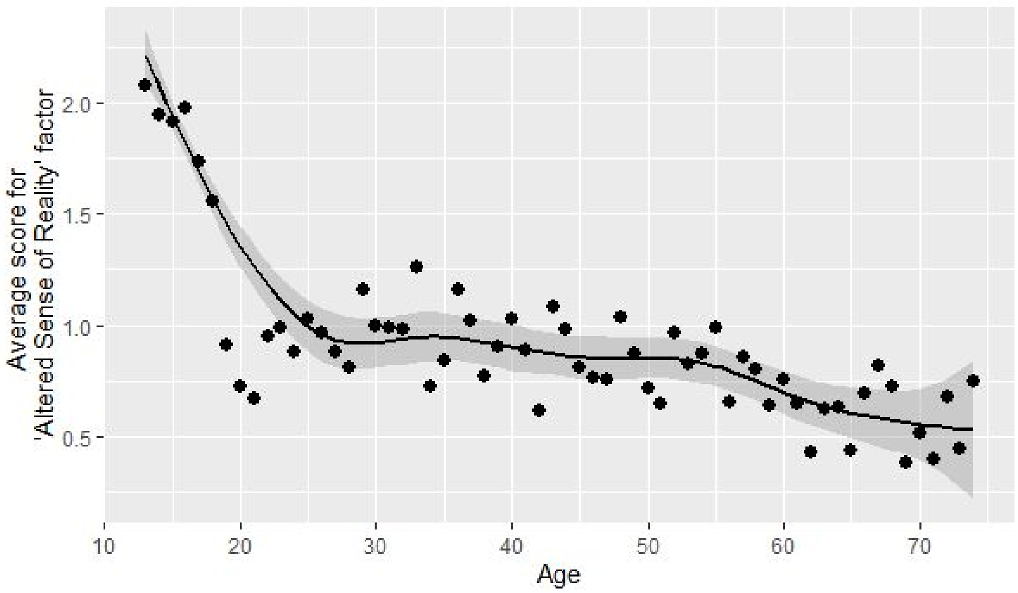
Average score for
'Altered Sense of Reality' factor
2.0
1.5
1.0
0.5
10
20
30
40
50
60
70
Age

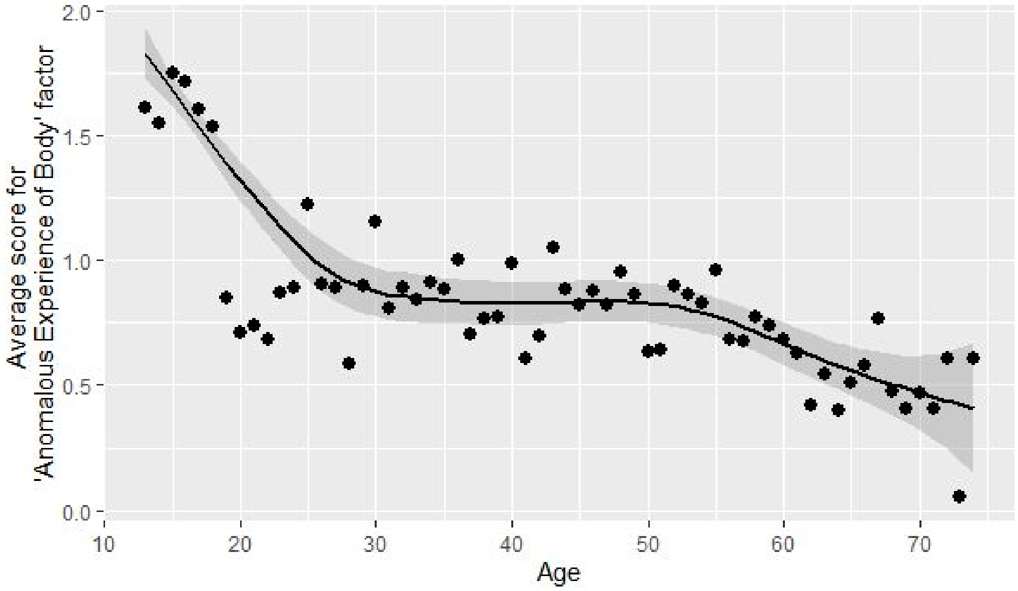
Average score for
'Anomalous Experience of Body' factor
2.0
1.5
1.0
0.5
0.0
10
20
30
40
50
60
70
Age

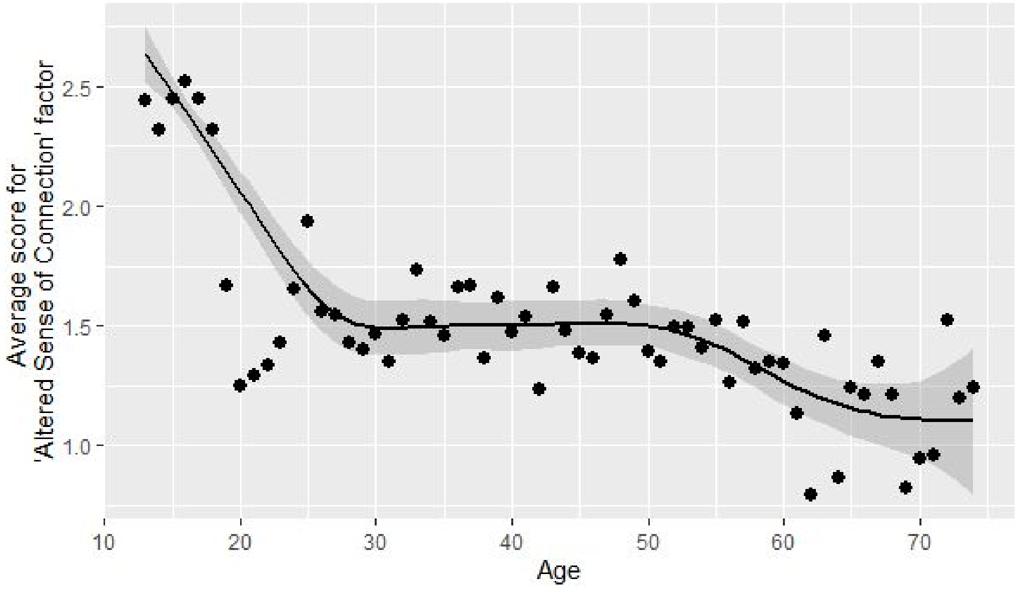
Average score for
'Altered Sense of Connection' factor
2.5
2.0
1.5
1.0
10
20
30
40
50
60
70
Age

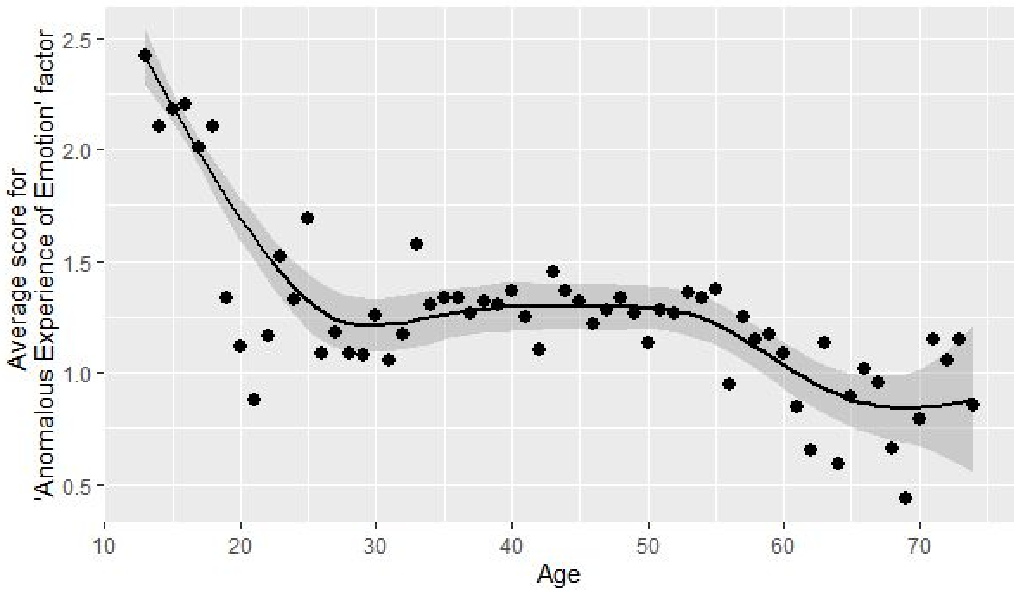
Average score for
'Anomalous Experience of Emotion' factor
2.5
2.0
1.5
1.0
0.5
10
20
30
40
50
60
70
Age

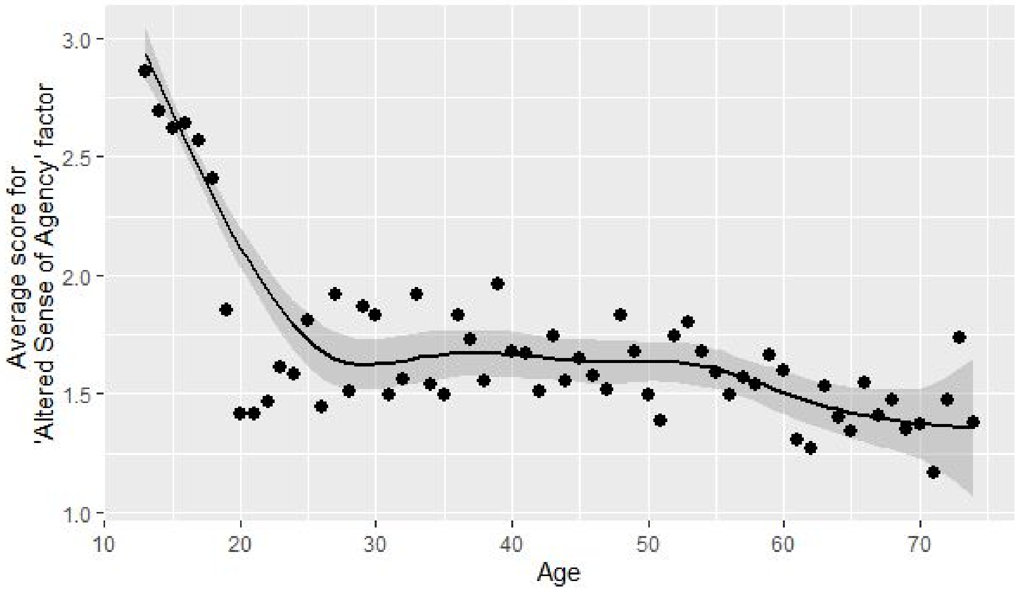
Average score for
'Altered Sense of Agency' factor
3.0
2.5
2.0
1.5
1.0
10
20
30
40
50
60
70
Age

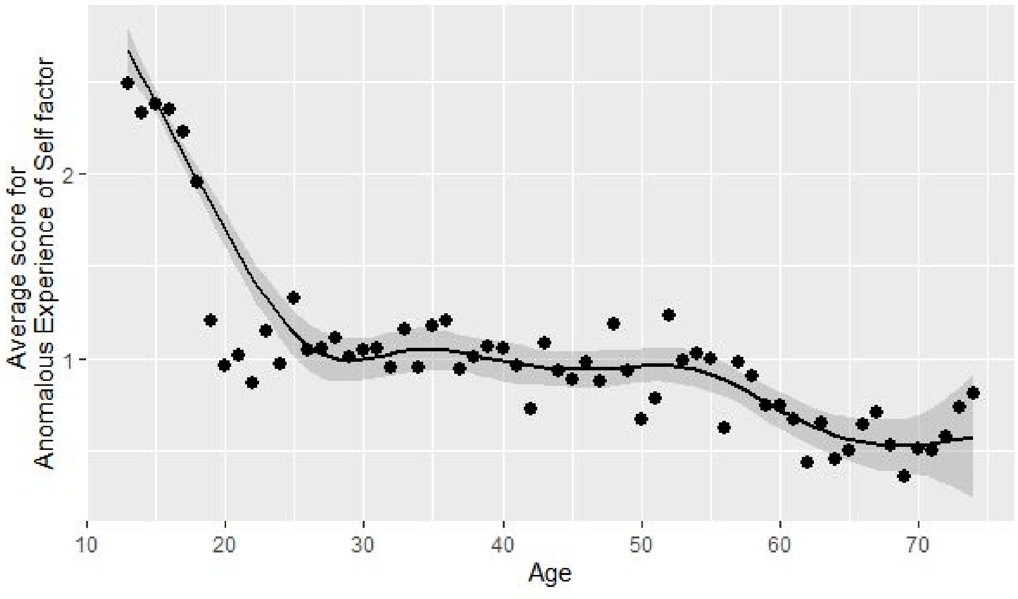
Average score for
Anomalous Experience of Self factor
2
1
10
20
30
40
50
60
70
Age

D. Model fit indices across age

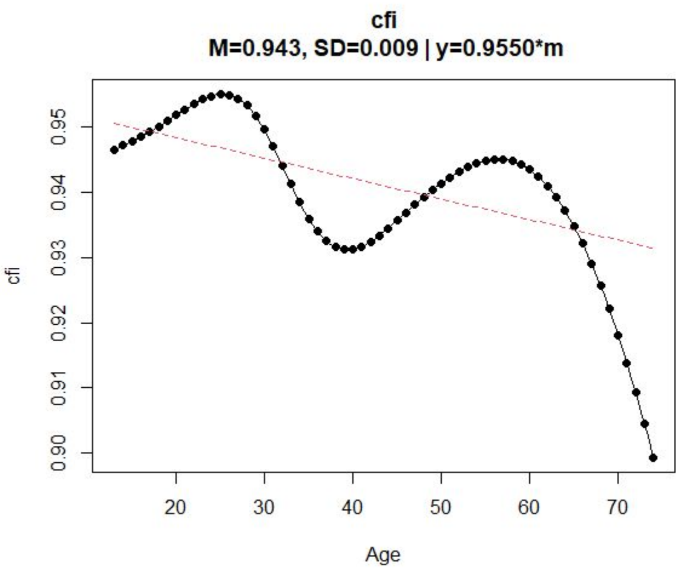

2a. Comparative Fit Index.

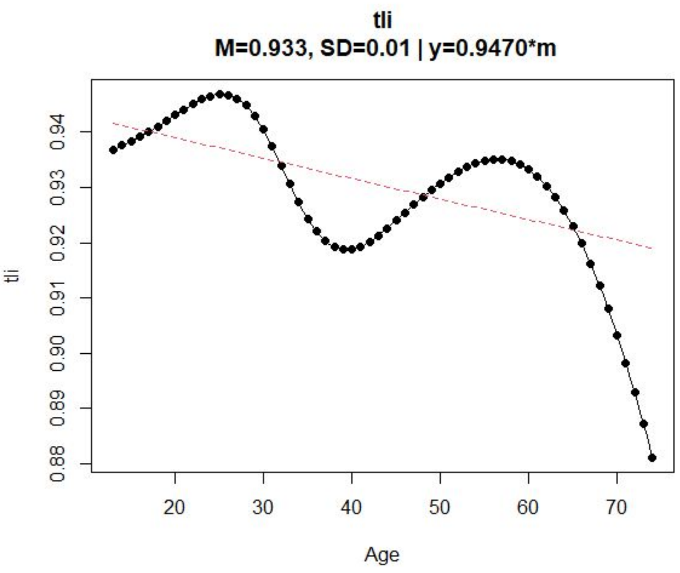

2b. Tucker-Lewis Index.

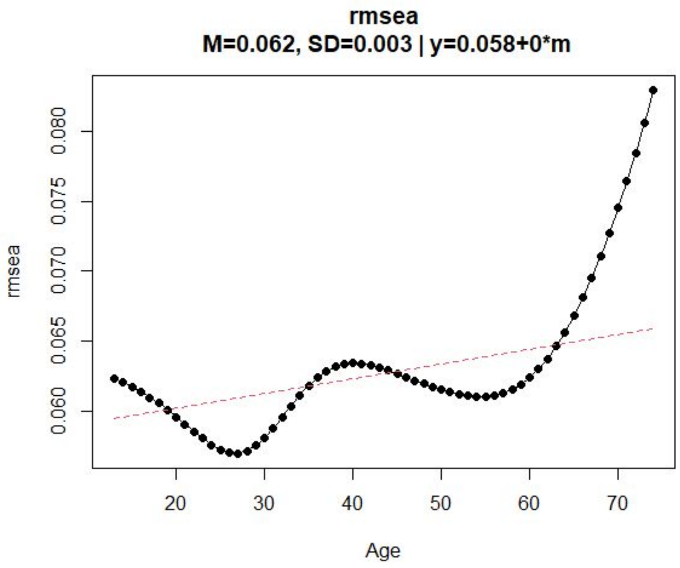

2c. Root Mean Square Error of Approximation.

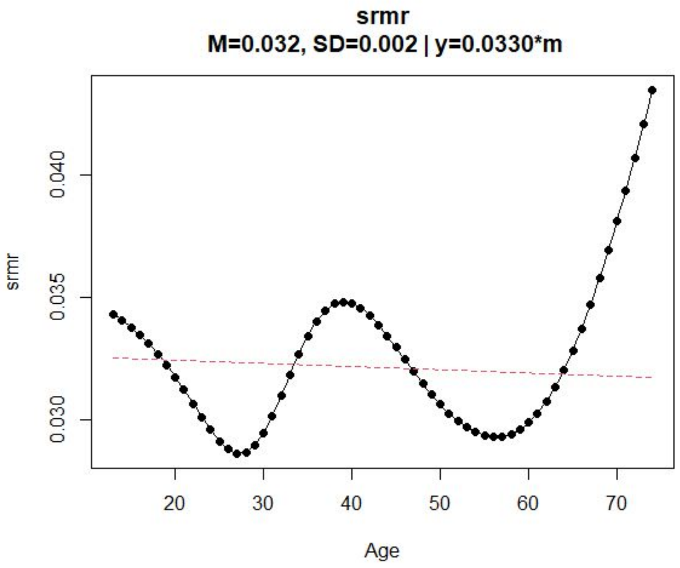

2d. Standardised Root Mean Square Residual.

Key:

M	*weighted average*
SD	*standard deviation*
y	*y-intercept*

E. Factor loadings for the items of the short-form (14 items) version of the ČEFSA scale

Factor	Item	Training confirmatory factor analysis	Validation confirmatory factor analysis
Anomalous Experience of the Self	8	1.000	1.000
	22	0.954	0.975
Anomalous Experience of the Body	16	1.000	1.000
	30	0.981	1.032
Altered Sense of Familiarity	24	1.000	1.000
	31	0.903	0.958
Anomalous Experience of Emotion	4	1.000	1.000
	18	1.120	1.117
Altered Sense of Connection	5	1.000	1.000
	12	0.988	0.964
Altered Sense of Agency	6	1.000	1.000
	13	1.129	1.060
Altered Sense of Reality	28	1.000	1.000
	34	1.027	0.996

NB: Item numbers and factor names are those used in the original (35-item) ČEFSA.
